# Medico-legal considerations in post-mortem imaging data: governance, ethics, and evidential use

**DOI:** 10.1093/bjro/tzag002

**Published:** 2026-01-20

**Authors:** Natasha Davendralingam, Susan C Shelmerdine, Amy-Lee Brookes, Imogen Jones

**Affiliations:** Department of Radiology, Tameside & Glossop NHS Foundation Trust, Ashton-under-Lyne OL6 9RW, United Kingdom; Anubix Ltd., London HP14 3FE, United Kingdom; Anubix Ltd., London HP14 3FE, United Kingdom; Department of Radiology, Great Ormond Street Hospital for Children NHS Foundation Trust, London WC1N 3JH, United Kingdom; UCL Great Ormond Street Institute of Child Health, London WC1N 1EH, United Kingdom; NIHR Great Ormond Street Hospital Biomedical Research Centre, London WC1N 1EH, United Kingdom; Royal Preston Hospital, Lancashire Teaching Hospitals NHS Foundation Trust, Preston, Lancashire PR2 9HT, United Kingdom; School of Law, The Liberty Building University of Leeds, Leeds, West Yorkshire LS2 9JT, United Kingdom

**Keywords:** autopsy, post-mortem, radiology, computed tomography, medicolegal, data protection, data privacy

## Abstract

Post-mortem imaging, in particular CT (PMCT), is increasingly used for death investigation in England and Wales, yet unlike “live” clinical imaging, this data falls outside traditional health-record legislation, creating uncertainty around data ownership, access rights, and disclosure obligations. This review examines the current data governance landscape surrounding post-mortem imaging data, identifying critical gaps requiring national guidance. We explore fundamental questions of data control between coroners and commercial service providers, noting how the absence of standardized frameworks has resulted in substantial regional variation in practice. Key challenges include inconsistent approaches to data storage, whether on clinical or dedicated PACS systems, varying data-retention periods, and disparate policies for third-party access by researchers, legal teams, and bereaved families. The evolving role of radiologists as expert witnesses in coronial and criminal proceedings presents additional complexities, particularly regarding who is best placed to explain imaging findings in court. We propose recommendations including national standards for data governance, standardized contractual frameworks clarifying data-controller relationships, protocols for secure storage and access controls, and defined competencies for radiologists presenting evidence in legal settings. Establishing robust governance foundations for post-mortem imaging data is essential to ensure this technology serves the public interest effectively, while maintaining legal defensibility and ethical integrity.

## Introduction

Post-mortem imaging (PMI) has become a transformative tool in some modern death investigations, offering a non or minimally-invasive alternative to autopsy while providing detailed anatomical visualization. Techniques include CT, micro-CT, MRI, ultrasound (US), and plain radiography (XR), all contributing valuable diagnostic and evidential information across adult and paediatric cases.

As the use of PMI expands across England and Wales, with some areas now performing nearly all examinations through minimally-invasive techniques, medico-legal challenges have emerged.^1–3^ These include variation in data governance, legal frameworks, and ethical standards. PMI generates sensitive data, yet existing law and regulation focus on living patients, leaving coroners and providers to interpret governance responsibilities.

Amongst many potential uses, PMI data can serve as primary evidence in legal proceedings, has the potential to reduce reliance on invasive autopsies, and can inform important medical research. However, without clear frameworks, researchers encounter inconsistent data, and coroners use their discretion inconsistently. This review examines the medico-legal landscape of PMI in England and Wales, addressing data control, ownership, evidential use, and governance gaps.

Underpinning this review is a commitment to simultaneously ensure that PMI is of evidential value, whilst minimizing the potential for distress and indignity associated with unnecessary invasive autopsies. We further wish to ensure that families do not face barriers to access which may compound their grief. Recent national reviews have highlighted the urgency in addressing these issues. The *Voicing Loss* study (ICPR, 2024) exposed the distress, confusion, and lack of transparency experienced by bereaved families within coronial processes, calling for greater compassion, clarity, and consistency in post-death care.^4^ Similarly, the *Fuller Inquiry—Phase 2 Report* (2025) identified profound deficiencies in post-death regulation, including the lack of regulation when external PMI services are engaged. This oversight is described as “partial, ineffective and, in significant areas, completely lacking,” with the Inquiry recommending the establishment of a statutory framework and a Commissioner for the Dignity of the Deceased.^5^ Together, these findings reinforce the urgent need for coherent, ethical, and legally robust governance of PMI and related practices across England and Wales.

## Legal frameworks for PMI

Under the Access to Health Records Act 1990, the term “health records” applies only to medical data collected during an individual’s lifetime, resulting in different legal frameworks applying to access of data for the living and the dead.^6^^,^^7^ This means that imaging conducted post-mortem falls outside the scope of most health regulations and are instead considered forensic data.

The duties of coroners to investigate deaths are set out in the Coroners and Justice Act 2009.^8^ Imaging ordered as part of this process is evidence within a legal investigation, not part of a health record. PMI may also be utilized in other contexts, for example in a “consent” hospital PM or where a family pays for a private PMI scan. Each purpose raises different legal questions. Key issues include control of the data and how it is accessed and used.

### Legal authority and data control

Clinical imaging data has 2 key parts. First, the images themselves. Second is their interpretation, including any written report. Together these form the relevant data. Especially where private services are engaged by the public sector (whether that be the NHS or the coroner) the storage and processing of the data can introduce complexity.

Coroners have the authority to commission post-mortem examinations and determine how the resulting data is used, stored, or disclosed. The Coroners (Investigations) Regulations 2013 further reinforce this, with Regulation 27 obliging coroners to maintain records of their investigations, including imaging data. The coroner pays for the various services involved in obtaining this data, and as such will have contractual arrangement with each party involved (and/or their employer).^9^

The Coroners and Justice Act 2009 provides coroners with discretion regarding how to carry out investigations within their area. This means that several different models are used nationally,^8^ each with varying schemes for access to scanners, radiologists, and the storage of data.^9^ For example, the Coroner’s Statistics report from 2024 for England and Wales stated that 19 coronial areas did not use PMI, which stands in stark contrast to the Lancashire and Blackburn with Darwen area where 88% of their PMs were carried out using only less invasive techniques such as PMI.^2^^,^^3^^,^^10^

The inconsistent use and models for the use of PMI in medico-legal death investigations can produce challenges and would benefit from clarification. In particular, disputes may arise when external private companies are involved to acquire, store, and report the imaging. The companies may view themselves as stakeholders with some degree of control, particularly if proprietary technologies/intellectual property are involved. The distinction between the data controller (the coroner) and the data processor (the company) becomes crucial. The coroner determines the purpose and means of processing the data. The company is bound by the terms of its contract and cannot make independent decisions about its use.

Once a person has died, most legal duties restricting access to health records no longer apply. Justice may demand a right of access (eg, coroners and medical examiners have a statutory right of access under the Access to Health Records Act 1990).^11^ However, access is still subject to ethical and some legal constraints. For example, a personal representative of the estate of the deceased (a person holding the Grant of Probate or Letters of Administration) can request access to a health record. The circumstances under which this can be withheld or redacted are set out in that Act.

### Research ethics, confidentiality, and access

Inconsistency of approach amongst coroners may create significant barriers to research. Some coroners may be more inclined to allow anonymized data to be used for research purposes, while others might be hesitant. Currently, this is a matter for them as the data controller.

In addition to informing medico-legal death investigations, PMI data has the potential to inform research that could improve future health outcomes and understanding of disease. Whilst any death can be distressing for the next-of-kin, we would suggest that improving future health outcomes as well as understanding individual deaths can be important for both society and the bereaved. Research can improve understanding and interpretation of PMIs, improving scope for avoiding unnecessary invasive autopsies. A careful balancing of interests and views, including increased efforts to inform the bereaved of the benefits, is therefore crucial if PMI data is to be best utilized.

Use and/or publication of any images for the purposes of research engages ethical as well as access issues. Together, the NHS and the learned societies provide a framework which could be usefully adapted by coroners dealing with requests to access PMI data for research. In addition to the ethical obligation to maintain confidentiality the NHS Health Research Authority provides a framework for balancing ethical concerns with public benefit.^12–14^

Confidentiality when dealing with PM data is less regulated than for the living, but it is no less ethically significant. While the General Data Protection Regulation and the Data Protection Act 2018 apply only to the living, the NHS Code of Confidentiality and guidance from the General Medical Council emphasize the need to handle PM data with sensitivity and respect.^15^ This extends to protecting the dignity of the deceased and considering the potential impact on their families.

We would note that research may be carried out and/or funded by non-state actors. In this context, access to PMI data might, for example, be sought to evaluate the technology which underpins a commercial interest in the provision of PMI services. Commercial gain introduces an additional ethical consideration, but this must not detract from the principles underpinning the framework, in particular the need to align health research with the public interest. Furthermore, as highlighted by the Fuller Inquiry,^5^ there is a need to ensure that private providers of PMI are held to the same standards when engaging with the deceased so as to protect their dignity.

There are several other circumstances in which external parties may wish to access PMI data. These engage different concerns, but there are commonalities in terms of the potential ethical issues and lack of consistent approach or guidelines.

Parties include:

- Legal teams in a variety of legal arenas (criminal, family, civil, and parties to inquest for example).
- Insurance companies investigating claims.
- The bereaved.

Control, access, and conditions for this will depend on individual context. For example, the Coroner retains control of images taken for a coronial investigation, but we would expect a Coroner to grant access to the bereaved for specific purposes, that is, an insurance claim. Access would remain subject to ethical constraints, including confidentiality, the strength of the justification, and the potential emotional and/or health benefits from disclosure.^16^ Where a scan is carried out privately for the next-of-kin, storage and access to the images are a matter of contract between the parties.

A key issue is the levying of charges for access; if requesting access to data held by external commercial company, there may be a cost associated with granting access (although access to records held by the NHS must be given free of charge).^11^ We would suggest that costs should not exceed those incurred in providing access (ie, not be profiteering), as commercial gain has already been costed into the initial contract for services. Of particular concern would be charges to the bereaved, who are vulnerable and can often be overwhelmed by the post-death administration process, especially where a death is unexpected or contentious.

These issues of access and consent directly influence how PMI data are later stored, secured, and presented in legal proceedings.

*Recommendations*

1. Establish a national framework to standardize access, governance, and ethical oversight of PMI data across coronial, research, and clinical contexts.
2. Define clear legal and contractual responsibilities for coroners as data controllers and service providers as data processors, ensuring transparency in ownership, control, and use of PMI data.
3. Embed robust consent and confidentiality safeguards, applying the ethical principles of the Human Tissue Act 2004—oversight, informed consent, and public benefit to all PMI data use, including research.
4. Mandate anonymization or pseudonymization of PMI data wherever possible, protecting privacy while enabling legitimate research and audit.
5. Adopt consistent professional and technical standards—guided by the Society of Radiographers and the joint RCR-RCPath statement, to uphold dignity, ethical integrity, and evidential reliability in PMI practice.
6. Create unified national governance and oversight structures, jointly endorsed by the Ministry of Justice, Human Tissue Authority, and relevant professional bodies, to ensure accountability and resolve disputes.

Together, these measures would harmonize legal and ethical standards across jurisdictions, providing clarity for coroners, families, and researchers alike.

## Governance and presentation of PMI data

The storage, management, and evidential use of PMI data raise critical questions regarding data governance, ethical handling, cybersecurity, and legal accountability. While hospital clinical PACS (Picture Archiving and Communication Systems) are widely used, their deployment for PMI requires additional safeguards because coronial data sit outside conventional NHS information-governance frameworks.

### Terminology and regulatory context

International guidance, including that of the *International Association of Forensic Radiographers (IAFR)* and the *Society of Radiographers (SOR)*, defines forensic imaging broadly as the acquisition of imaging for any legal or investigative purpose, whether involving the living or the deceased.^1^^,^^17^ This umbrella term includes CT, MRI, post-mortem CT angiography (PMCTA) enahcradiography, ultrasound, and selected external-examination photography.

In England and Wales, however, an administrative distinction has developed between “forensic” and “coronial” PMI. The former is commonly used to refer to police-led or suspicious-death investigations overseen by *Home Office Registered Forensic Pathologists (HORFPs)*, whereas the latter refers to coroner-commissioned examinations of non-suspicious or unexplained deaths. As legal scholars have noted, this divide is largely procedural rather than conceptual: both forms of PMI serve a legal purpose, generate evidential material, and may ultimately be presented in court.^1^^,^^17^

To avoid reinforcing an artificial separation, we use the term *PMI* throughout this article to describe coronial practice in England and Wales, while acknowledging that it forms part of the wider forensic-imaging continuum recognized internationally.

In accordance with the Human Tissue Authority (HTA) guidance updated October 1, 2025, unenhanced PMCT or other imaging performed without contrast administration is classified as non-invasive.^18^ However, enhanced PMCT, including PMCTA and PMCTV, involves the introduction of perfusion contrast media and is therefore considered minimally invasive and an HTA-licensable activity.^18^ This distinction replaces previous generic references to PMCT as “invasive” and aligns PMI practice with current UK regulatory definitions.^1^^,^^18–21^

### Storage and access of PMI data

#### Shared clinical PACS vs dedicated PACS for deceased data

Clinical PACS platforms, whether in NHS or private environments, are technically robust, incorporating access controls, audit trails, and role-based permissions. However, they were designed to manage images of living patients governed by health-record legislation. When repurposed for PMI, additional legal and ethical considerations arise that reflect not flaws in the systems themselves, but the distinct legal status of deceased-person data.

According to the Information Commissioner’s Office (ICO), UK GDPR applies only to living individuals and therefore does not cover data relating to the deceased. Nonetheless, such data remain protected under other laws and duties—including common-law confidentiality, the Freedom of Information Act (FOIA), and specific coronial or police oversight; and must be handled carefully, particularly where third-party information appears within the record.^2^^,^^22–27^ This creates a regulatory space in which PMI images and reports require bespoke governance rather than standard clinical policies.

Dedicated PMI PACS or encrypted cloud environments can offer operational advantages: clear segregation from clinical workflows, customized metadata (eg, case numbers rather than NHS identifiers), and restricted access for coronial or forensic teams.^2^^,^^23–27^ However, these systems entail additional infrastructure and licensing costs and are not mandatory.

A balanced approach is to retain PMI data within existing PACS where feasible, provided that governance measures include:

- Visual identifiers distinguishing PMI from clinical studies.
- Role-based access limited to authorized users.
- Routine audit-trail and cyber-risk reviews.
- Defined retention and deletion policies approved by the coroner or relevant data controller.

Equivalent standards should apply across public and private providers, with data-retention and access policies modelled on NHS and coronial frameworks to ensure parity and accountability.

#### Cybersecurity considerations

Cybersecurity vulnerabilities are an increasing concern across healthcare networks, and the risk landscape is even less regulated within PM services. Unlike clinical imaging, which falls under NHS Digital, Data Security and Protection Toolkit (DSPT) compliance, and national cyber-governance frameworks, PMI data currently lack any equivalent regulatory oversight. Most PMI services operate through mixed NHS-coronial or private partnerships, resulting in varied governance responsibilities and inconsistent technical safeguards.

UK media reports have highlighted persistent NHS infrastructure weaknesses and outdated PACS systems that leave sensitive data exposed to cyberattack.^23–25^ While these issues apply broadly to clinical radiology, PMI datasets may be even more vulnerable because they fall outside the scope of GDPR, lack clear data-controller designation, and are not subject to routine cybersecurity auditing or patch-management policies.

Nguyen *et al* reported that healthcare remains one of the most frequently breached sectors globally, with PACS environments among the most targeted for ransomware and unauthorized access. Between 2014 and 2023, more than 510 million healthcare records were compromised worldwide, many through vulnerabilities in radiology networks.^24^ Common weaknesses include unencrypted DICOM transfers, outdated operating systems, and inadequate user-access control.^24^

Given that PMI services often rely on shared or locally configured PACS without central oversight, similar vulnerabilities may exist but remain largely undocumented and unregulated. To mitigate risk, national and institutional policy should prioritize:

- Regular cybersecurity audits and prompt patch management of PACS/DICOM systems.
- End-to-end encryption of data transmission and storage, including within hospital or coronial networks.
- Multi-factor authentication and the principle of least privilege for all PMI users.
- Alignment of cybersecurity and data-protection standards between NHS, coronial, and private PMI providers, following NHS Digital and ICO guidance.

Framing these safeguards as best-practice recommendations, rather than criticisms, acknowledges the capability of existing infrastructure while recognizing that PMI operates in a more fragmented regulatory environment. Establishing explicit cybersecurity expectations for PMI will strengthen public trust, protect sensitive coronial data, and prevent future medico-legal vulnerabilities.

#### Retention and disclosure of PMI data

Significant variability persists in retention and disclosure practices across England and Wales. Some coroners stipulate retention periods contractually, while others delegate discretion to providers. Such inconsistency affects legal readiness and the long-term availability of PMI data for inquests or appeals.

Release of PMI images and reports to third parties (families, legal representatives, insurers) requires explicit coroner authorization, as suggested by professional guidance.^1^^,^^2^^,^^19^^,^^21^

Although external-examination photography is not routinely performed across the UK, the same underlying governance principles apply. PMI data are managed under coronial authority in the same way as other forms of non-invasive documentation, such as body-map or descriptive records prepared by pathologists or anatomical pathology technicians. Disclosure and access are authorized by the coroner under the *Coroners and Justice Act* rather than regulated through the *Human Tissue Act 2004*.

### Use of PMI findings in legal proceedings

Post-mortem imaging findings are increasingly presented within coronial inquests and, in some instances, criminal and civil proceedings. The evidentiary admissibility of PMI is well established; courts routinely accept radiological findings as valid forms of expert evidence, analogous to clinical imaging used in life. However, the degree of reliance and presentation practices vary geographically, reflecting differences in training and procedural familiarity rather than any inherent legal uncertainty.^2^^,^^3^ In several jurisdictions, PMI has been accepted as the sole investigation underpinning the cause of death.^2^^,^^28^ More commonly, courts regard PMI as supportive rather than definitive, particularly in the absence of histology or toxicology.

The key evidential issue is not admissibility but consistency and accountability, ensuring that PMI evidence is interpreted and presented by appropriately trained experts using transparent methodology. Clear national protocols for how PMI findings are prepared, labelled, and archived for legal use would strengthen both reliability and public trust.

Responsibility for presenting PMI findings varies between service models. In pathologist-led jurisdictions (eg, Leicester, Oxford, London), pathologists usually present both imaging and autopsy findings, citing radiology reports as supporting evidence.^2^^,^^3^ In radiologist-led or hybrid models (eg, South Manchester, Lancashire), radiologists themselves present PMI findings, particularly where imaging is the primary investigation.^3^^,^^28^

The central question is not who presents the evidence, but whether those giving evidence possess the necessary imaging literacy, legal understanding, and awareness of modality limitations. Courts require clarity regarding methodology, provenance, and interpretation limits. Standardizing expectations for competence and documentation would promote uniform practice across coronial and criminal jurisdictions.

Beyond determining who presents the findings, it is critical that radiologists undertaking PMI reporting possess appropriate training in forensic interpretation and medico-legal procedure. Conventional diagnostic training does not routinely address artefacts of decomposition, injury interpretation, or evidentiary documentation standards. Structured subspecialty training, through accredited fellowships, professional-body guidance, and expert-witness programmes, is therefore essential for ensuring reliability and confidence in testimony. Equally, pathologists require foundational imaging literacy to integrate radiological findings effectively. Developing this dual competence fosters a genuinely collaborative model in which radiology and pathology complement, rather than duplicate, one another.

Current national and international guidelines acknowledge the need for training in PM interpretation but stop short of defining minimum competency standards. The *Royal College of Radiologists* and *Royal College of Pathologists* joint guideline (G182, 2021) advises that radiologists must be appropriately trained and aware of PM artefacts and death-related changes, yet it provides no specification regarding training duration, case exposure, or accreditation benchmarks.^19^ Similarly, the *ISFRI Best Practice Standards* (2025) emphasize that physicians interpreting PMI “should be appropriately trained and experienced” but do not set quantitative thresholds.^17^ In practice, there is wide variation across UK providers. In one private coronial service, radiologists are required to complete a structured 6-month virtual fellowship (including theoretical and practical assessment), double-report at least 50 supervised cases, and participate in ongoing peer-review auditing. Conversely, some services appear to permit independent reporting following only brief observational exposure (approximately 0.5-3 days). Although these examples are based on current practice rather than formal published standards and are therefore anecdotal, they illustrate substantial variability in expectations and preparedness. This lack of consistency underscores the need for nationally agreed minimum training requirements, accreditation pathways, and audit frameworks for PMI reporting.

While formal training for presenting evidence in coronial or criminal courts is not currently mandatory, it should be actively encouraged for clinicians involved in PMI reporting. Forensic pathologists in the UK routinely receive courtroom-presentation training, and expert-witness courses and certifications are increasingly available to medical professionals. Such preparation does not question clinical competence; rather, it improves understanding of what the medico-legal system requires—clarity, transparency, and effective communication. For PMI reporters, this knowledge enhances the quality and defensibility of testimony, facilitates collaboration with coroners and legal teams, and helps clinicians protect themselves when appearing in coronial, criminal, or civil proceedings.

*Recommendations*

1. Establish national governance standards for the storage, retention, and secure handling of PMI data, ensuring clear segregation from clinical systems and compliance with data-protection principles.
2. Standardize technical and evidential protocols for image labelling, metadata, and archiving to prevent misidentification and maintain the integrity of PMI findings in legal contexts.
3. Formalize training and accreditation pathways in forensic interpretation, medico-legal reporting, and courtroom presentation for radiologists and pathologists involved in PMI.
4. Promote collaborative models of testimony and quality assurance, enabling radiologists and pathologists to present integrated evidence supported by regular audit and peer review.
5. Implement robust cybersecurity and data-protection requirements, harmonized with NHS Digital and Information Commissioner’s Office (ICO) standards, to safeguard coronial and forensic datasets.

Collectively, these measures would ensure that PMI practice in England and Wales remains legally defensible, ethically robust, and operationally consistent while fostering public confidence in its evidential reliability.

## Special paediatric considerations

It is widely accepted that respecting the dignity of the deceased, including in early developmental stages, is crucial.^29^ This extends to how images are acquired, stored, and used. While the general principles of coronial and forensic imaging apply for children as they do for adults, the vulnerability of this population, coupled with the emotional and societal sensitivities in relation to paediatric death, necessitates a nuanced approach.

One primary distinction lies in the legal status of stillborn foetuses. In many jurisdictions, including the UK, a miscarried/stillborn foetus is not considered a legal person, meaning that many legal frameworks, such as those governing health records and data protection for living individuals, also do not directly apply.^2^ This can create ambiguity regarding data ownership, access, and confidentiality. The absence of a clear legal person status for stillborns can impact how their imaging data is handled, particularly concerning parental rights to access or control such information. The Royal College of Obstetricians and Gynaecologists (RCOG) Green-top Guidelines on the care of late intrauterine foetal death and stillbirth primarily focus on clinical management and investigation, acknowledging the profound impact on parents.^30^ Similar guidelines exist from the Royal College of Pathologists for performing autopsy (including minimally-invasive techniques like imaging).^31^^,^^32^ However, these do not address medico-legal implications of data access, handling, or sharing.

The emotional distress of parents experiencing stillbirth or the death of a child means that transparency and sensitivity in handling imaging data are essential. The potential for images to be used for research or educational purposes, while valuable for advancing knowledge, must be balanced against the need to protect privacy and avoid re-traumatization of families. Anonymization and pseudonymization are key strategies, but the unique nature of some paediatric and foetal imaging (which may include rare congenital diseases) still carry a risk of re-identification, requiring robust safeguards which should be considered as detailed above.

*Recommendations*

1. Create a unified governance framework that defines data ownership, parental access, and confidentiality in paediatric and foetal PMI, aligning coronial and healthcare standards while safeguarding dignity and compassion.
2. Implement ethical and consent safeguards for any secondary use of paediatric or foetal imaging data, ensuring transparency with bereaved families and balancing research benefit with the need to minimize distress.
3. Strengthen collaboration and data-protection measures through anonymization, pseudonymization, and multidisciplinary oversight by radiologists, pathologists, obstetricians, and ethicists to address both legal and emotional complexities.

Together, these recommendations would safeguard dignity, enhance ethical governance, and provide compassionate, consistent handling of imaging data in cases involving children and stillbirths.

## Conclusions

The absence of national standards leaves PMI in an ambiguous position—straddling healthcare, law, and technology, yet governed fully by none. This gap has resulted in uneven practice, fragmented data control, and inconsistent evidential standards. PMI’s growing role in modern death investigation means that addressing this is of increasing importance.

To fulfil its potential as both a scientific and civic tool, 3 priorities must now be addressed:

- Governance—establish national frameworks for secure data storage, retention, and access.
- Accountability—clearly define the respective duties of coroners and providers through standardized contractual models.
- Competence—embed structured, reciprocal training in imaging interpretation, ethics, and courtroom presentation.

These reforms are not bureaucratic; they are fundamental to justice. Embedding PMI within a coherent ethical and regulatory framework will safeguard the dignity of the deceased, will minimize the unnecessary re-traumatization of the bereaved whilst also taking account of their interests, and the evidential reliability upon which public trust depends.

Ultimately, the future of PMI depends on collaboration—between coroners, clinicians, radiographers, radiologists, pathologists, technologists, and policymakers, united by a single aim: to transform digital precision into conclusions that are defensible in law and compassionate in practice.

## Data Availability

Data sharing is not applicable to this article as no datasets were generated. Summary of findings from previously published articles are provided already in figures within this review.
